# Upscaled Skeletal Muscle Engineered Tissue with In Vivo Vascularization and Innervation Potential

**DOI:** 10.3390/bioengineering10070800

**Published:** 2023-07-04

**Authors:** Vladislav Borisov, Laia Gili Sole, Gregory Reid, Giulia Milan, Gregor Hutter, Martin Grapow, Friedrich Stefan Eckstein, Giuseppe Isu, Anna Marsano

**Affiliations:** 1 Laboratory of Cardiac Surgery and Engineering, Department of Biomedicine, University of Basel, 4031 Basel, Switzerlandlaia.gilisole@unibas.ch (L.G.S.); giulia.milan@unibas.ch (G.M.); friedrich.eckstein@usb.ch (F.S.E.);; 2 Cardiac Surgery, Department of Surgery, University Hospital Basel, 4031 Basel, Switzerland; grapow@herzzentrum.ch; 3 Laboratory of Brain Tumor Immunotherapy, Department of Biomedicine, University of Basel, 4031 Basel, Switzerland

**Keywords:** scale-up, perfusion-bioreactor, tissue engineering, vascularization, innervation, skeletal myoblasts, SVF cells

## Abstract

Engineering functional tissues of clinically relevant size (in mm-scale) in vitro is still a challenge in tissue engineering due to low oxygen diffusion and lack of vascularization. To address these limitations, a perfusion bioreactor was used to generate contractile engineered muscles of a 3 mm-thickness and a 8 mm-diameter. This study aimed to upscale the process to 50 mm in diameter by combining murine skeletal myoblasts (SkMbs) with human adipose-derived stromal vascular fraction (SVF) cells, providing high neuro-vascular potential in vivo. SkMbs were cultured on a type-I-collagen scaffold with (co-culture) or without (monoculture) SVF. Large-scale muscle-like tissue showed an increase in the maturation index over time (49.18 ± 1.63% and 76.63 ± 1.22%, at 9 and 11 days, respectively) and a similar force of contraction in mono- (43.4 ± 2.28 µN) or co-cultured (47.6 ± 4.7 µN) tissues. Four weeks after implantation in subcutaneous pockets of nude rats, the vessel length density within the constructs was significantly higher in SVF co-cultured tissues (5.03 ± 0.29 mm/mm^2^) compared to monocultured tissues (3.68 ± 0.32 mm/mm^2^) (*p* < 0.005). Although no mature neuromuscular junctions were present, nerve-like structures were predominantly observed in the engineered tissues co-cultured with SVF cells. This study demonstrates that SVF cells can support both in vivo vascularization and innervation of contractile muscle-like tissues, making significant progress towards clinical translation.

## 1. Introduction

Loss of functional skeletal muscle is a pathological condition that can result from various causes such as trauma, chronic infection, tumor ablation, and open bone fractures [1,2,3]. When the muscle loss exceeds a certain threshold, the human body becomes unable to recover or compensate for the loss of soft tissue, leading to a condition known as volumetric muscle loss (VML) [4]. VML poses a significant medical challenge, and while traditional rehabilitation methods exist, regenerative medicine offers a promising treatment option [5]. Particularly in cases of massive VML injuries that do not respond to conventional therapies, exogeneous-mediated tissue augmentation could provide a solution [6,7]. Tissue engineering has made significant advancements in this field with numerous studies exploring the efficacy and safety of different approaches using bioactive materials and functionalized scaffolds [8,9,10,11,12,13,14]. However, these methods often have limitations such as fast resorption rates and incomplete integration within the surrounding healthy tissue [15]. Alternative strategies involve the transfer of autologous tissue [6,16,17,18] with the associated problems of donor site morbidities, or the generation of in vitro engineered flaps [19,20,21,22] with often relatively small sizes and thicknesses only up to 1 mm. This study aims to contribute to the existing data by investigating the in vitro engineering of skeletal muscle tissues with clinically relevant masses and sizes.

One of the main challenges in tissue engineering is achieving sufficient tissue thickness without impairing tissue function. Skeletal muscle myoblasts, for example, require a nearby source of oxygen no further than 150 µm for their proliferation and differentiation [23]. Perfusion-based bioreactors have been proposed as a solution, providing a constant supply of nutrients and oxygen, waste removal, and beneficial mechanical stimuli [24,25,26,27]. However, generating functional mm-thick engineered tissues able to maintain cell viability during implantation and surgical procedures remains a challenge [28]. There are two main approaches to address this issue [29]: the pre-vascularization of engineered tissues through biomimetic microvascular networks [12,30,31], or the stimulation of host vessel infiltration in vivo [32,33,34]. Vascularization is crucial not only for cell survival but also to meet the high metabolic demands of regenerating skeletal muscle tissue. Additionally, host nerve endings are essential to prevent muscular atrophy [35]. The innervation of engineered muscle tissues has been explored through various methods such as surgical transfer of a neurotized bundle [36], or co-culture with murine-derived motor neurons [37] or human progenitor neural cell lines [38]. The incorporation of adult stromal cells in the engineered skeletal muscle-like tissues offers a promising strategy due to their angiogenic, neurogenic, and anti-apoptotic properties [39,40,41]. In this regard, the stromal vascular fraction (SVF) cells isolated from fat tissue are an excellent candidate compared to other cell types, such as adipose- or bone marrow-derived mesenchymal stem cells (MSCs). Indeed, SVF cells display reduced related morbidities and, importantly, enriched regenerative potential due to their highly heterogeneous composition [42,43].

In a previous study, small-scale (8 mm-diameter) functional skeletal muscle-like tissues of a relevant thickness (3 mm) were successfully generated using a perfusion-derived unidirectional flow [44]. This work aims to generate an upscaled version of 50 mm-diameter and 3 mm-thick engineered muscle-like constructs with muscle-like features and vascularization and innervation potential. For the first time, this study combines murine-derived skeletal muscle myoblasts as a proof-of-principle parenchymal source with human adipose-derived SVF cells as a heterogenous mesenchymal source with enhanced regenerative potential. The hypothesis is that the co-culture of SVF cells will enhance the angiogenic and neurogenic potential of the upscaled 3D skeletal muscle-like engineered tissue.

## 2. Materials and Methods

### 2.1. In Vitro Studies

#### 2.1.1. Skeletal Myoblast Culture

Primary skeletal myoblasts (SkMbs) were isolated from both forelimb and hindlimb muscles of neonatal (2–5 day old) C57BL/6 mice (Jackson Laboratory), as previously described [45]. Briefly, the muscles were minced and digested with 1.5 U/mL of collagenase D (Roche Molecular Biochemicals, Basel, Switzerland), 2.4 U/mL of dispase II (Roche Molecular Biochemicals), and 2.5 mm CaCl_2_ at 37 °C. Isolated cells were pre-plated for 45 min on 0.1% calf skin collagen-coated (Sigma–Aldrich, St. Louis, MO, USA) dishes to allow for fibroblast adhesion. Non-adhering myoblasts were collected and seeded on a new collagen-coated dish. The SkMb proliferating medium consisted of low glucose Dulbecco’s modified Eagle’s medium (DMEM) (Sigma–Aldrich) supplemented with 38.5% F-10 nutrient mix (Sigma–Aldrich), 20% fetal bovine serum (FBS) (HyClone, Thermofisher, Reinach, Switzerland), 1% penicillin/streptomycin, 1% L-glutamine, 1% HEPES, and 2.5 ng/mL fibroblast growth factor-2 (FGF-2) (R&D Systems, Minneapolis, MN, USA). Upon 70% confluency, cells were detached with 0.25% (*w*/*v*) trypsin/0.1% (*w*/*v*) EDTA. Reagents were purchased from ThermoFisher Scientific, Reinach, Switzerland unless otherwise noted.

#### 2.1.2. Stromal Vascular Fraction Cell Isolation

Adipose tissue was obtained from three healthy donors undergoing elective plastic surgery after informed consent and according to a protocol approved by the Ethics Committee of the University Hospital Basel. All investigations conformed to the declaration of Helsinki. Adipose tissue was minced and digested with 0.075% type II collagenase (355 U/mg, Worthington Biochemical Corporation, Lakewood, NJ, USA) in phosphate-buffered saline (PBS) under continuous shaking for 60 min at 37 °C. After centrifugation at 1500 rpm for 10 min, the lipid-rich layer was discarded, and the cellular pellet was washed with PBS. Fibrous debris resulting from the digestion was removed by filtering the cell suspension through a 100 µm and 70 µm nylon mesh. The resulting SVF cells were resuspended in culture medium consisting of high glucose DMEM (Sigma–Aldrich) supplemented with 10% FBS (HyClone), 1% penicillin/streptomycin, 1% L-glutamine, and 1% HEPES. Cell pellets were frozen in 10% dimethylsulfoxide (DMSO) and 90% FBS and stored in liquid N_2_. For experiments, SVF cells were quickly thawed in a 37 °C water bath and immediately used. Cell survival rate was between 70% and 90%.

#### 2.1.3. Cell Seeding

Disc-shaped pieces 50 mm in diameter were cut from 3 mm-thick collagen type I sponges (Avitene^TM^ Ultrafoam^TM^ haemostat, Davol Inc., Tiverton, RI, USA) and soaked overnight in culture medium at 37 °C. For cell seeding purposes, 1.05 × 10^8^ SkMbs, corresponding to a density of 2.2 × 10^7^ cells/cm^3^, were re-suspended in 1.8 mL culture medium. For co-culture studies, 6.9 × 10^7^ SVF cells, corresponding to 1.46 × 10^7^ cells/cm^3^, were further added. The resulting cell suspension was seeded onto the pre-wet sponges with the help of a multi-channel pipette, ensuring a complete distribution of the cells throughout the whole surface area. Sponges were placed in an agarose-coated Petri dish to prevent cell adhesion to the plastic and kept inside a cell culture incubator for two hours before transferring them to the perfusion-based bioreactor.

#### 2.1.4. Perfusion-Based Bioreactor Culture

For the direct perfusion of medium through the cell-seeded scaffolds, a previously developed electropolished AISI 316L stainless steel bioreactor (Applikon Biotechnology BV, Delft, The Neatherlands) was used [25]. Four inlet and four outlet ports ensured a uniform perfusion of the medium over the entire surface area of the scaffold. Inside the chamber, the 50 mm-diameter cell-seeded scaffold was secured between two stainless steel wire grids (50 mm-diameter) (Bopp AG, Zürich, Switzerland) and clamped by a 3 mm-thick and 2.5 mm-wide Teflon ring, producing an inner perfused scaffold cross-section of 45 mm-annular diameter. On the inflow side of the bioreactor, each of the four inlets was connected to a separate channel (exactly the same length and size) mounted on a multi-channel peristaltic pump (Reglo Digital MS-4/8, Ismatec SA, Glattbrugg, Switzerland). The flow rate of each inlet was set to 7.5 mL/min, resulting in a perfusion flow rate of 30 mL/min through the whole scaffold, based on a previous study [25]. The flow rate was chosen to promote even cell distribution throughout the entire construct. Scaffolds were perfused with culture medium for either 6, 9, or 11 days for monoculture studies and for 11 days for co-culture studies with SVF cells under a standard incubation environment of 37 °C and at 5% CO_2_. Assembling of the scaffold within the bioreactor was performed under a cell culture aseptic environment. The culture medium was changed every 3 days.

### 2.2. In Vivo Study

#### Subcutaneous Implantation

Subcutaneous pockets on the back of nude rats were chosen as a suitable in vivo implantation model to obtain a proof-of-principle of the angiogenic and neurogenic potential of the generated patches, as previously described [42]. Animals were treated in compliance with Swiss Federal guidelines for animal welfare, and all procedures were approved by the Veterinary Office of the Canton Basel (Basel, Switzerland) (license number: 2608_30631). After 11 days of perfusion culture, discs of 8 mm-diameter were biopsied from the inner and most central part of each engineered construct (4 independent discs from one construct for each mono- or co-culture experimental group). Three male nude athymic eight-week-old rats (Hsd: RH-rnu/rnu, Envigo Gmbh, Horst, The Neatherlands) underwent general anesthesia by inhalation of isoflurane (1.5% to 3% volume) in O_2_ (0.6 L/min). Constructs were implanted in subcutaneous pockets created through incisions of 2 cm to 3 cm length on the dorsal musculature (4 constructs/rat/experimental group). Two cell-free collagen sponges were pre-wet in culture medium for 24 h and implanted as controls. Animals were sacrificed after 28 days by CO_2_ inhalation. Scaffolds were explanted and further processed for histological evaluation.

### 2.3. Analysis

#### 2.3.1. Permeability Test

The hydraulic permeability of each construct was determined after static seeding and at the end of in vitro culture as a measure of scaffold remodeling. The hydraulic permeability (*k*) of the porous scaffold was evaluated starting from Darcy’s law (Equation (1)), which allows the permeability to be expressed as a function of (i) the culture medium mass flow rate (*ρM*), (ii) the scaffold cross-sectional area (*A*), (iii) the scaffold thickness (∆x), and (iv) the pressure gradient across the scaffold (∆P):

(1) $$k=\frac{\rho M}{A}\frac{∆x}{∆P}$$

Starting from this mathematical formulation (Equation (1)) and considering the thickness and the cross-sectional area of the scaffold as constants, it was possible to determine the hydraulic permeability by measuring the flow rate for a fixed ∆P. To impose a constant ∆P, a sufficiently large reservoir was placed at a 25 cm height difference from a bottom reservoir, generating a constant head pressure of 2.45 kPa. The change of hydrostatic pressure during the experiment due to the drop of liquid level in the upper reservoir was assumed to be negligible. In order to consider the non-negligible distributed and concentrated losses along the hydraulic connections, Darcy’s law was coupled to Bernoulli’s equation [46], resulting in a corrected formulation of the hydraulic permeability (Equation (2)), where $M_{B1}$ is the mass flow rate through the system without any porous scaffold, $M_{B2}$ is the mass flow rate through the disc-shaped scaffold, and *r* is the outlet section radius. As a result, the hydraulic permeability was obtained as an indirect measurement by the evaluation of the flow rates *M_B1_* and *M_B2_*. The permeability of cell-free collagen scaffolds was used as a control.

(2) $$k=\frac{∆x}{A\cdot M_{B2}}\times \frac{2\pi^{2}r^{4}}{{\left(M_{B1}/M_{B2}\right)}^{2}-1}$$

#### 2.3.2. Histology and Immunofluorescence

In vitro engineered patches were cut in 6 radial equal pieces, 3 of which were dedicated to histology (Figure S1A). Samples were fixed overnight in 4% formaldehyde and further cryopreserved for 24 h in 30% sucrose prior to embedding in optimal cutting temperature (OCT) compound (CellPath). Frozen blocks were sectioned at 10 µm with a cryostat (Bopp AG). As a general histo-morphological evaluation, sections were stained with hematoxylin and eosin (H&E) according to standard protocols. For immunofluorescence staining, slides were treated for 1 h in blocking buffer consisting of 0.3% Triton X-100 in PBS supplemented with 5% goat serum and 2% bovine serum albumin (Sigma-Aldrich) followed by 1 h of incubation with the following primary antibodies: rabbit polyclonal anti-myosin heavy chain (Myh3) (M7523, Sigma-Aldrich) at 1:150; rabbit polyclonal anti-Ki67 (15580, Abcam, Cambridge, UK); mouse monoclonal anti-Human Nuclei (HuNu) (MAB1281, Millipore, Burlington, MA, USA); mouse monoclonal anti-PECAM-1 (CD31) (119339, Abcam) at 1:100; mouse monoclonal anti-pan-neurofilament marker (NF) (837801, Biolegend, San Diego, CA, USA); mouse monoclonal anti-Myogenic Differentiation 1 (Myod) (SPM427, Novusbio, USA); mouse monoclonal anti-Myogenin (Myog) (5FD, Novusbio, Littleton, CO, USA); mouse monoclonal anti-α-sarcomeric actinin (AB9465, Abcam); and α-bungarotoxin, Alexa Fluor™ 488 conjugate (B13422, Invitrogen, Thermo-Fisher Scientific, Waltham, MA, USA). Subsequently, tissue sections were incubated for 1 h with secondary antibodies labelled with Alexa Fluor^®^ 488, 546, or 647 dyes (Invitrogen). Nuclei were stained using 4′,6-diamidino-2-phenylindole dihydrochloride (DAPI) (Invitrogen) at 0.125 mg/mL in blocking buffer. Antibodies were diluted in blocking buffer at 1:200 unless otherwise written. All bright field and immunofluorescent images were acquired with an automated BX63 (Olympus Deutschland GmbH, Hamburg, Germany), Ti_2_ 257 (Nikon, Tokyo, Japan), or CSU-W1 (Nikon) microscope.

#### 2.3.3. Quantification Based on Image Analysis

##### Maturation and Fusion Indexes

The index of fusion was obtained by counting the number of nuclei in the Myh3-stained structures (counting structures with at least 2 nuclei) and expressed as a percentage of the total number of nuclei in the analyzed field of view. The maturation index was instead calculated as the percentage of Myh3-stained myotubes with five or more nuclei to the total number of myotubes [47]. For co-culture studies, maturation and fusion indexes were calculated after subtracting the number of human cells (double positive for HuNu and DAPI). Image analysis was performed with ImageJ 1.51n software (Research Service Branch, NIH).

##### Proliferation Index

Cell proliferation activity was defined as the ratio of Ki67^+^ nuclei to all DAPI-stained nuclei. For images from co-culture studies, the proliferation of SkMbs and SVF cells was calculated separately. The number of proliferating SVF cells was defined as simultaneously positive nuclei for Ki67, HuNu, and DAPI. The proliferation index for SkMbs was acquired by subtracting the number of nuclei simultaneously positive for Ki67, HuNu, and DAPI from all Ki67 and DAPI double positive nuclei. Image analysis was performed with ImageJ 1.51n software (Research Service Branch, NIH, USA).

##### Cell Distribution and Density

Quantification of the cell distribution in vitro was performed by dividing the samples dedicated to histology into three further regions: periphery, middle, and center (Figure S1). The periphery was defined as the area along the radius of 2.1 mm length × 3 mm thickness (corresponding to 3 images acquired with a 20× objective), measured from the edge of the construct; the center was defined as 2.1 mm from the geometrical center of the construct; the sample left between the center and the periphery was defined as the middle. The number of all nuclei stained by DAPI was divided by the analyzed area (µm^2^). For the co-culture condition, the number of HuNu^+^ cells was subtracted from the total DAPI-stained nuclei, thus giving only the number of SkMbs. Image analysis was performed with ImageJ 1.51n software (Research Service Branch, NIH, USA).

##### In Vivo Vessel Length Density

Wide field 20× immunofluorescent images of in vitro and in vivo slides were acquired with a BX63 microscope (Olympus). Vessel length density (VLD) was calculated as the length of all CD31^+^ vessel-like structures divided by the area of the analyzed field (mm/mm^2^). All measurements were performed with CellSens software (version 2.1, Olympus).

##### Analysis of Neurofilament-Positive Structures

Wide field 20× immunofluorescent images of in vivo slides were acquired with a Ti_2_ microscope (Nikon). Image analysis was performed using open-source software for digital pathology (QuPath, version 0.4.0) [48]. NeuroFilament (NF)^+^ cells were quantified and showed as percentage of the total amount of cells (DAPI-positive cells). Organized NF^+^ structures were also quantified and presented according to the number of cells that composed them. Structures were formed by a minimum of 3 cells. Round organized nerve-like (NL) structures were further analyzed for their cell number content and area.

#### 2.3.4. Contractility Test

The contractile force generated from in vitro engineered tissues was assessed in a custom-built bioreactor [49]. Briefly, rings of 8 mm outer diameter and 6 mm inner diameter were punched out of mono- and co-culture constructs after 11 days of perfusion and placed between two nitinol rods within the bioreactor. Nitinol rods were moved apart until the disc shape construct became visibly stretched, but not as much as bending would occur. Ring-shaped constructs were exposed to external electrical pacing with rectangular pulses of 10 ms duration, 100 mA amplitude, and frequencies increasing from 1 Hz up to 7 Hz. Krebs–Henseleit 1× buffered solution equilibrated with carbogen gas (95% O_2_; 5% CO_2_) at 37 °C was used as medium. The contraction force generated from engineered tissues was calculated from videos acquired upon electrical stimulation, as previously described [49]. Briefly, 4× videos were algorithm-processed to quantify the deflection of the nitinol rods and correlate it to the force exerted by the engineered constructs as a function of the cantilever bending stiffness. Functionality was assessed in duplicates for each condition.

#### 2.3.5. Statistical Analysis

For the monoculture condition, the following independent experiments were conducted: two for 6 days, three for 9 days, and two for 11 days. For the co-culture condition, due to the limit of the number of available freshly isolated SVF cells, only one and two independent experiments were performed for 9 and for 11 days, respectively. All image quantifications were performed from at least 18 images from 4 different slides of every independent sample. All data are presented as mean ± standard error of the mean (SEM), unless otherwise stated. One-way or two-way ANOVA tests were used for normal populations. For all other graphs, non-parametric Mann–Whitney tests and Kruskal-Wallis tests were used for single and multiple comparisons, respectively. Statistical significance was set at *p* values < 0.05. Data were processed with GraphPad Prism software, version 9.

## 3. Results

### 3.1. Bioreactor Description and Experimental Plan

**Figure 1:**
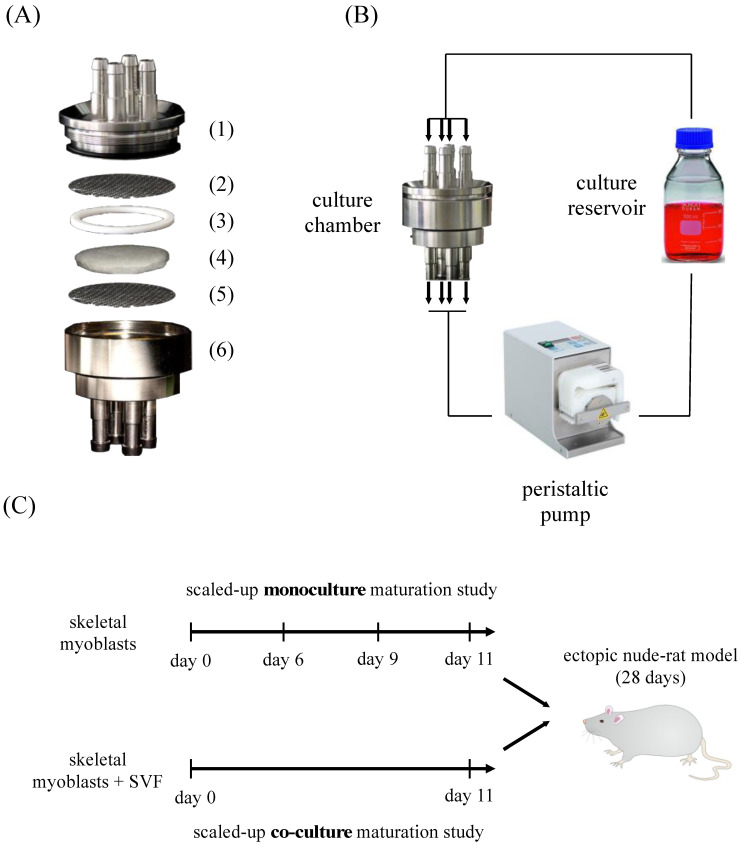
Design of the perfusion-based bioreactor and experimental plan. (**A**) The bioreactor chamber was composed of two stainless-steel components (1, 6) in which a collagen-based scaffold (4) fixed by a Teflon ring (3) and two metal grids (2, 5) was placed. (**B**) Schematic representation of the perfusion bioreactor connected to the culture reservoir and the peristaltic pump. (**C**) Experimental plan: monoculture engineered tissues generated in 6, 9, or 11 days; co-cultured engineered tissues were generated in 11 days. Monoculture and co-culture constructs generated in 11 days were further implanted for 28 days into subcutaneous pockets of nude rats.

The generation of scaled-up skeletal muscle engineered tissues of a clinically relevant size was supported by the use of a perfusion-based bioreactor (Figure 1A). This was composed of a two-component stainless steel chamber with four inlet and outlet ports to allow culture media to uniformly flow over the 50 mm-diameter scaffold [25]. Homogeneous perfusion throughout the large area of the scaffold was further ensured by clamping it with a Teflon ring. Additionally, two stainless steel wire grids were positioned in between the bioreactor chambers and the scaffold to mechanically support it and avoid any flow-derived deformations. Upon static seeding of the cells, the assembled bioreactor was connected to a closed unidirectional perfusion circuit composed of a peristaltic pump and a media reservoir (Figure 1B). Scaled-up maturation in vitro studies of SkMbs were performed for 6, 9, or 11 days. In parallel, the effect of SVF cells on the generation of SkMb-based engineered tissues was evaluated in co-culture maturation studies for 11 days. Long-term in vitro engineered tissues (11 days) were implanted into subcutaneous pockets of male nude rats for 28 days (Figure 1C).

### 3.2. Cell Distribution and Proliferation in In Vitro Monoculture System

**Figure 2:**
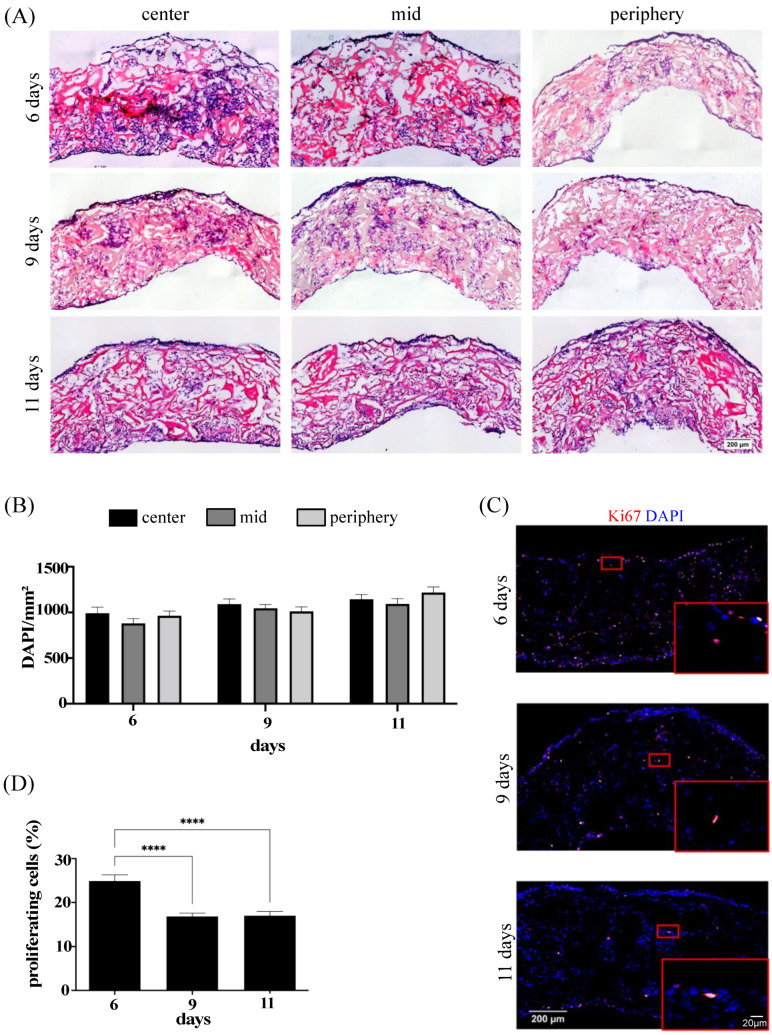
In vitro distribution and proliferation of SkMbs. (**A**) H&E representative images of the center, the mid, or the periphery of monoculture-based tissues engineered at 6, 9, or 11 days. Scale bar = 200 µm. (**B**) Quantification of the cell distribution (DAPI/mm^2^) at 6, 9, and 11 days for the three different analyzed regions (center, mid, and periphery). (**C**) Immunofluorescence images stained for DAPI and Ki67. Scale bar = 200 µm. High magnifications are indicated by red boxes (scale bar = 20 µm). (**D**) Quantification of proliferating cells (% of Ki67^+^). Cell distribution and cell proliferation were assessed from three different samples from two independent experiments at day 6; three independent experiments at day 9; and two independent experiments at day 11 (**** *p* < 0.0001).

To verify cell distribution and proliferation following in vitro perfusion culture, histology and immunofluorescence analyses were performed throughout the 50 mm-diameter and 3 mm-thick collagen-based mesh. Three pieces representative of the center, the middle, and the periphery of the large-scale construct were sampled and further processed for histological analyses. As shown by H&E staining (Figure 2A), SkMbs appeared to be uniformly distributed across the entire scaffold area.

A significant number of cells was clearly visible in the outermost edge and also between the empty pores of the scaffold, where newly formed ECM was deposited. These observations were further confirmed by quantifying the density of DAPI^+^ cells (presented as DAPI/mm^2^, Figure 2B). Results showed similar values of cell density in the center, the middle, and the periphery of the scaffold for the three analyzed time points. It is well established that upon muscle injury, activated myoblasts, also known as myogenic precursors or satellite cells, exit their quiescent state and enter several rounds of proliferation prior to exiting the cell cycle to further differentiate into myocytes and fuse together into myofibers, the building blocks of complex skeletal muscle structures [50]. Therefore, the number of proliferating SkMbs throughout the culture time was also investigated by immunostaining for the proliferation marker Ki67 (Figure 2C). After 6 days of in vitro maturation culture, more than 20% of the SkMbs were still undergoing proliferation (24.91 ± 1.38%), while these levels significantly decreased after 9 and 11 days (16.85 ± 0.74% and 17.02 ± 0.93%, respectively) (Figure 2D).

### 3.3. Skeletal Muscle Cell Maturation in In Vitro Monoculture System

**Figure 3:**
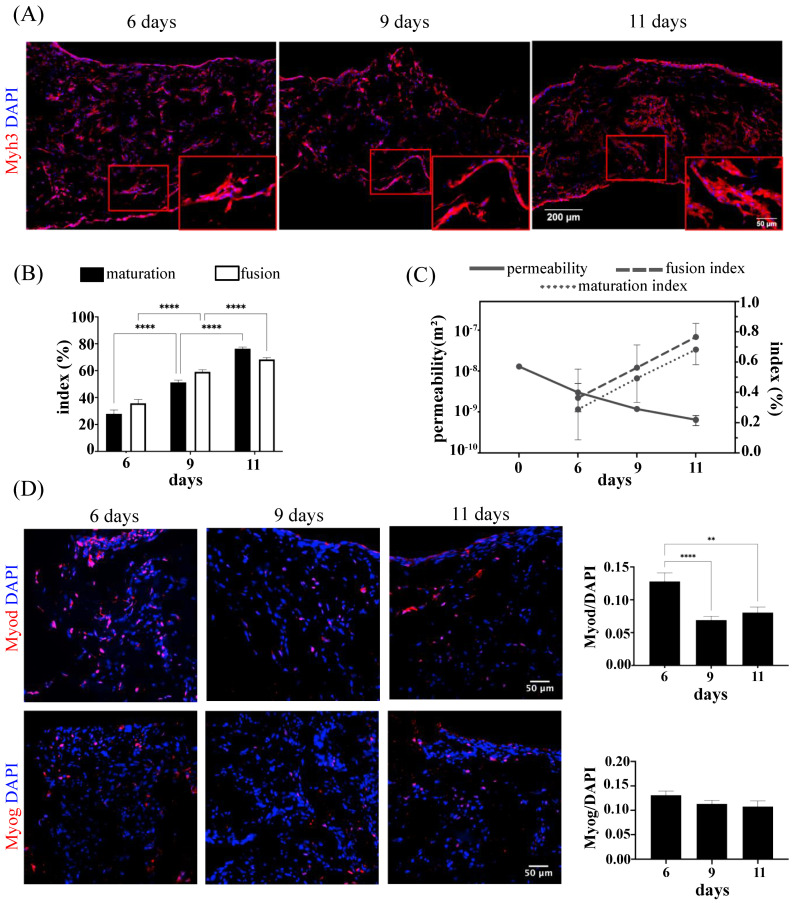
In vitro differentiation and maturation of SkMbs. (**A**) Representative images of samples stained for Myosin Heavy Chain 3 (Myh3) and DAPI for the three analyzed time points. Scale bar = 200 µm. High magnifications are indicated by red boxes (scale bar = 50 µm). (**B**) Quantification of the indexes of maturation and fusion were calculated based on Myh3 staining and expressed as percentage over total cells (DAPI^+^). (**C**) Correlation plot between the measured permeability (m^2^) and the indexes of maturation and fusion. Permeability was calculated on the whole construct from two independent experiments for each time-point. Indexes of maturation and fusion were assessed from 3 different samples from two independent experiments at day 6; three independent experiments at day 9; and two independent experiments at day 11. Data are presented as mean ± SD. (**D**) Representative images of staining for transcription factors Myod and Myog and their quantification over DAPI^+^ cells, performed from three different samples from two independent experiments for each analyzed time-point (** *p* < 0.01, **** *p* < 0.0001). Scale bar = 50 µm.

Proliferation and differentiation of SkMbs are tightly coupled processes, whereby the latter is initiated only when the first one is ceased. The generation of mature skeletal engineered tissues was further confirmed by immunostaining for myosin heavy chain (Myh3), a muscle structural protein and a hallmark of differentiation into fibers [51]. Immunofluorescence staining for Myh3 showed that upon 6 days of culture, the majority of SkMbs was positive for the Myh3, indicating their commitment to differentiation (Figure 3A). The effect of the in vitro culture time on the efficiency and the extent of SkMb differentiation was further evaluated by two parameters: the index of maturation, and the index of fusion. The index of maturation was calculated as the percentage of myotubes (positive for Myh3) with ≥5 nuclei. The index of fusion was instead obtained by counting the number of nuclei in the Myh3-stained structures (counting structures with at least two nuclei) and expressed as a percentage over the total number of nuclei in the analyzed field of view. Compared to day 6, both maturation and fusion indexes significantly increased after 9 and 11 days of culture, indicating that SkMbs underwent a process of differentiation and fusion into myotube-like structures (Figure 3A,B).

Hydraulic permeability of the engineered tissues was monitored throughout the different culture times as a correlation to the size and the structure of the pores within the scaffold [52]. Permeability was measured in scaffolds perfused with culture medium at 37 °C and assuming the viscosity of the medium and water was very close [53]. In our experimental set-up, the hydraulic permeability for cell-free collagen scaffolds was quantified as 1.29 × 10^−8^ m^2^ (1.29 × 10^−11^ m⁴/Ns), resulting in values close to previous studies on the permeability of collagen type I sponges [54]. After 6 days of perfusion, permeability dropped to 2.98 × 10^−9^ ± 1.97 × 10^−9^ m^2^ and further decreased up to 1.18 × 10^−9^ ± 4.95 × 10^−11 ^m^2^ and 6.42 × 10^−10^ ± 1.74 × 10^−10^ m^2^ at day 9 and 11, respectively. As expected, permeability negatively correlated with the indexes of maturation and fusion, proving evidence of the increased degree of tissue complexity obtained throughout the culture time (Figure 3C).

Additional immunofluorescence staining was performed for Myod and Myog (Figure 3D). Myod is a skeletal muscle-specific transcription factor activated early during myogenic commitment of SkMbs [55]. The expression of Myod (calculated as the ratio of Myod positive cells over the total number of cells in a field of view) was drastically reduced from day 6 to day 9 and 11 (0.13 ± 0.013, 0.070 ± 0.006, and 0.08 ± 0.008, respectively). In the case of Myog, another transcription factor restricted to skeletal muscle with an important role in the terminal differentiation of SkMbs and their maturation into myofibers [56], no significant differences could be observed between day 6 (0.13 ± 0.008), day 9 (0.11 ± 0.007), and day 11 of culture (0.11 ± 0.011).

### 3.4. Cell Distribution and Proliferation in In Vitro Co-Culture System

**Figure 4:**
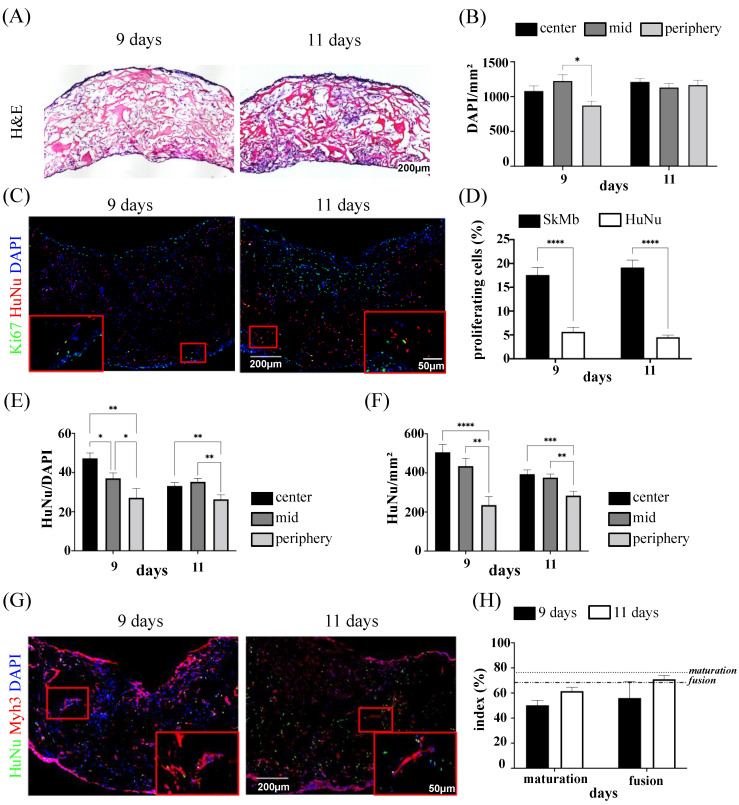
In vitro distribution, proliferation, and maturation of SkMbs in the SVF co-culture. (**A**) Representative image of H&E-stained slices from the center of SkMb engineered tissues co-cultured with SVF cells. Scale bar = 200 µm. (**B**) Cell distribution within the center, the middle (mid), and the periphery of the constructs at 11 days. (**C**) Representative image from immunofluorescent-stained slices for HuNu and Ki67 proliferation marker, scale bar= 200 µm. High magnifications are indicated by red boxes (scale bar = 50 µm). (**D**) Quantification of proliferating cells (Ki67^+^ cells). (**E**) Human cell density (HuNu/mm^2^) and distribution of HuNu^+^ cells over DAPI^+^ cells (**F**) within the different analyzed regions. (**G**) Representative immunofluorescence image stained for Myh3, HuNu, and DAPI. Scale bar = 200 µm. High magnifications are indicated by red boxes (scale bar = 50 µm). (**H**) Quantified indexes of maturation and fusion at 11 days in the presence of SVF cells. Horizontal lines from the *Y*-axis show the index of maturation and fusion calculated after 11 days of culture in the monoculture condition. Image analyses were performed from three different samples from two independent experiments for 11 days (* *p* < 0.05, ** *p* < 0.01, *** *p* < 0.001, **** *p* < 0.0001).

Prompt vascularization of engineered tissues upon in vivo implantation is vital to ensure survival of the cells and to prolong their therapeutic potential over time. To this end, full characterization of the patches generated by combining SkMbs with SVF cells, the latter being rich in mesenchymal cells but also in perivascular and endothelial cells, was performed after 11 days of co-culture prior to implantation. H&E staining of engineered constructs at 11 days unveiled a dense but homogeneous cell distribution (Figure 4A). The cell density after 11 days of culture was quite similar at the periphery, the center, and the middle of the construct (Figure 4B). SkMbs had significantly higher levels of proliferation compared to SVF cells after 11 days of in vitro culture (SkMb: 19.19 ± 1.5%, SVF: 4.29 ± 0.44%, *t*-test *p* < 0.0001) (Figure 4C,D). Noteworthily, SkMbs showed similar levels of proliferation to those observed during the monoculture upscaling studies (17.02 ± 0.93%) (Figure 2D). Towards the edge of the large construct, the density of SVF cells decreased at the periphery (Figure 4E). The quantification of the ratio between HuNu-positive cells over total DAPI also showed a significant reduction between the center and the other evaluated areas (Figure 4F).

We next aimed to investigate the effects, if any, of the SVF cells on the differentiation, maturation, and fusion of SkMbs into myotubes. To this purpose, immunofluorescence co-staining for HuNu and Myh3 was further performed (Figure 4G). Noticeable, SVF cells, detected by immunostaining for HuNu, predominantly occupied the internal pores of the scaffold, while SkMbs were more numerous on the free-edge surfaces (Figure 4G). The index of maturation at day 11 (61.59 ± 2.5%) was significantly lower compared to that obtained in the monoculture studies (76.63 ± 1.2%, Mann–Whitney test *p* < 0.0001). No significant differences were observed when comparing the index of fusion (71.03 ± 2.57%) at day 11 to that obtained from monoculture experiments (68.26 ± 1.38%) (Figure 4H).

### 3.5. Skeletal Muscle Cell Maturation in In Vitro Monoculture and Co-Culture System

**Figure 5:**
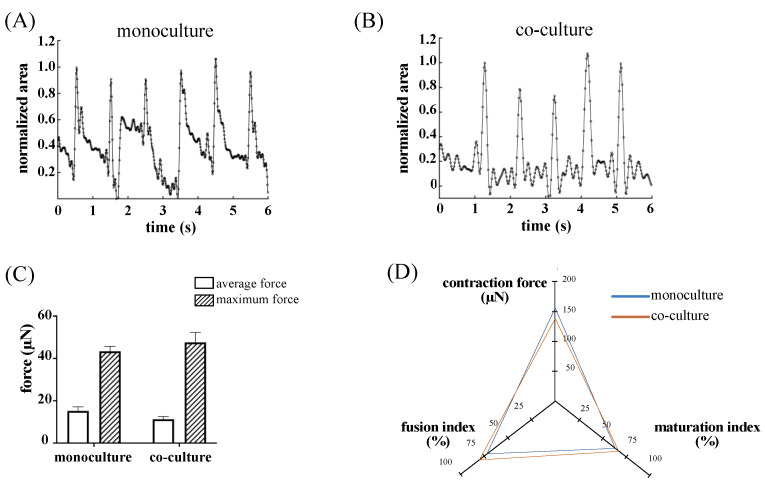
Functionality of engineered tissues. (**A**,**B**) The fractional area change (FAC) plots of monoculture and co-culture-based constructs, respectively, as a measure of generated contraction upon electrical pacing. (**C**) Calculated average and maximum force (μN). (**D**) Three-dimensional coordinate plot showing the correlation between the contraction force, the fusion index, and the maturation index in mono- and co-cultured tissues. The force of contraction was calculated from five independent rings sampled from one monoculture and one co-culture experiment.

Contractility of the large 3D skeletal muscle tissues was assessed by stimulating punched rings (8 mm and 6 mm outer and inner diameter, respectively) with electrical current pulses. The response to electrical pacing was calculated for monoculture and co-culture samples. In both cases, engineered constructs synchronized to the external electrical stimulation by contracting, as seen by the narrow high peaks in the fractional area change (FAC) plots (Figure 5A,B).

Average contractile forces developed upon electrical pace stimulation from SkMb monoculture-derived rings were similar to those obtained from co-culture tissues (15.21 ± 1.94 µN and 11.30 ± 1.22 µN, respectively). Likewise, the presence of SVF cells did not have an impact on the generated maximum force upon electrical pacing compared to monoculture samples (47.64 ± 4.71 µN and 43.42 ± 2.28 µN, respectively) (Figure 5C). Indexes of fusion and maturation, as well as contractility, are visualized together in Figure 5D as key parameters to assess the quality of maturation and function of the engineered constructs. The graph shows the overlapping of the functional parameters for mono- and co-culture engineered tissues, confirming that SVF cells do not impair the contractility and maturation or fusion capacity of the SkMbs.

### 3.6. In Vivo Integration and Neuro-Vascular Induction: Monoculture Versus Co-Culture System

**Figure 6:**
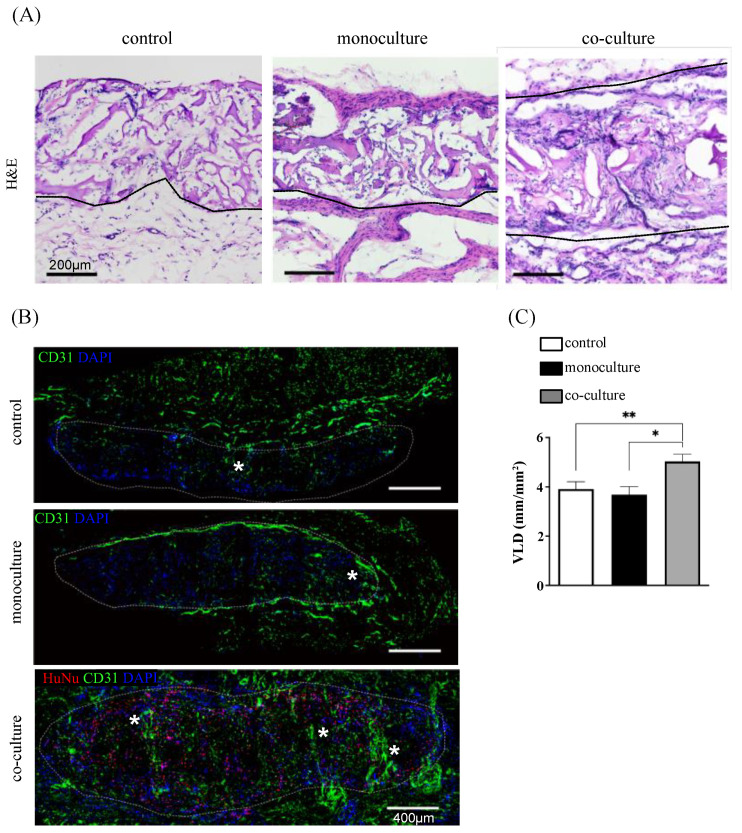
Integration, cell survival, and induction of angiogenesis in vivo. (**A**) Representative images of H&E staining in explanted tissues after 28 days of implantation. Scale bar = 200 μm. (**B**) Representative images of constructs stained for CD31 as a marker of endothelial cells for the evaluation of angiogenesis. Dotted lines indicate the perimeter of the implanted engineered constructs. Stars (*) indicate some of the blood vessels infiltrating the implants. (**C**) Vessel length density (VLD) quantification based on length of CD31-stained vessel-like structures (mm/mm^2^). Analyses were performed on four different images of the whole construct from four independent implanted constructs per condition (mono- or co-culture) (** *p* < 0.01). Scale bar = 400 μm. (**A**–**C**) Empty scaffolds represent the controls.

To assess whether the co-culture with SVF cells facilitates the ingrowth of newly-formed capillaries within the mm-thick construct, biopsies of 8 mm-diameter were sampled from in vitro generated skeletal muscle-like tissues cultured for 11 days, with or without the presence of SVF cells, and further implanted for 28 days into subcutaneous rat pockets. Histological images from H&E-stained slices showed that the structural integrity of the implanted scaffolds was maintained throughout the 4-week period. Indeed, areas belonging to the initially seeded collagen type-I scaffolds were still clearly visible in both monoculture and co-culture groups (Figure 6A). Implants were integrated within the surrounding tissue, and no fibrous-capsule formation was found. SVF cells were detectable within the engineered tissues, as observed by HuNu staining, confirming their retention (Figure 6B). Infiltration of vascular structures from the host was assessed by CD31 staining. The ingrowth of vessels within the scaffold was similar for both SkMb-engineered tissues (monoculture) and empty scaffolds (VLD: 3.91 ± 0.30 mm/mm^2^ and 3.68 ± 0.32 mm/mm^2^, respectively) (Figure 6C). In the co-culture condition, this was significantly enhanced up to 5.03 ± 0.29 mm/mm^2^, confirming the angiogenic potential of the SVF and thus suggesting its benefit to vascularize and further ensure the survival of mm-thick muscle-engineered tissues by facilitating efficient oxygen and nutrient transport.

**Figure 7:**
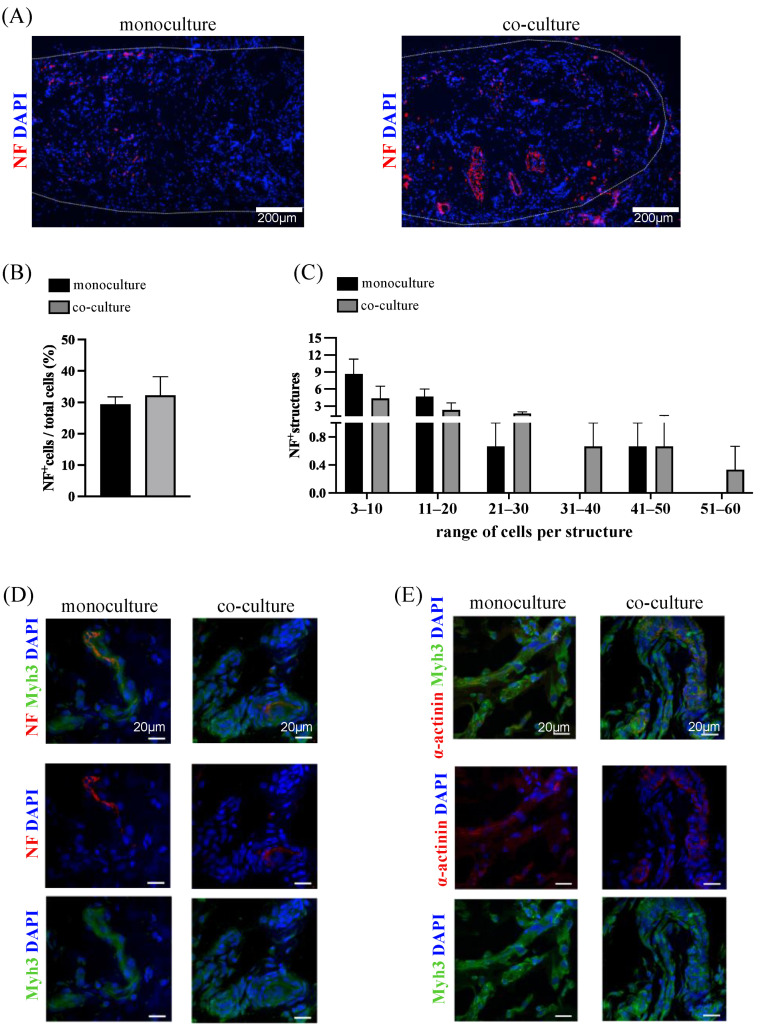
Induction of neurogenesis in vivo. (**A**) Immunofluorescence staining for NF in monoculture and co-culture. Scale bar = 200 µm. (**B**) Quantification of NF^+^ cells expressed as percentage of total amount of cells (DAPI^+^). (**C**) Analysis of NF^+^ structure complexity based on the number of NF^+^ cells included. (**B**,**C**) Eight fields in each of 3 monoculture constructs and of 3 co-culture constructs were analyzed. Representative images of monoculture and co-culture structures stained for Myh3 (green), NF (red), and DAPI (blue) (**D**) and for Myh3 (green), α-sarcomeric actinin (red), and DAPI (blue) (**E**). (**D**,**E**) Scale bar = 20 µm.

In addition to their angiogenic potential, adipose-derived SVF cells have been described to hold neurogenic and neuroprotective effects, mainly through the release of neurotrophic factors [40,57,58]. To investigate whether SVF cells promoted neurite outgrowth, possibly in response to paracrine-guided cues, we performed immunostaining for neurofilament (NF) protein, the major component of the neuronal cytoskeleton (Figure 7). In the presence of SVF cells, in vivo implanted constructs showed the noticeable presence of single organized neurofilament structures, suggesting a possible positive effect in inducing neurite outgrowth from recruited peripheral nerves. Indeed, the ingrowth of complex nerve-like structures was observed within the constructs and their surrounding tissue (Figure 7A).

Both the monoculture and the co-cultured constructs displayed similar percentages of NF^+^ cells (Figure 7B). However, the quantification of NF^+^ structures showed that in the monoculture group, the majority of organized cells were predominantly composed of a small number of cells (mainly in the 3–10 or 11–20 cell range) (Figure 7C). The co-culture constructs instead possessed the highest number of organized structures with a superior number of cells (namely, in the 21–30, 31–40, and 51–60 cell range) (Figure 7C). Highly organized round nerve-like structures, as presented in the co-culture in Figure 7A, were predominantly present in the presence of the SVF, as shown in Table 1 (1 and 7: number of round nerve-like structures in monoculture and co-culture, respectively). Furthermore, there was noticeable proximity between the NF^+^ cells and the elongated Myh3^+^ cells in both the monoculture and co-culture (Figure 7D). Furthermore, a neuromuscular junction (NMJ) with a typical bretzel-like shape was not observed in either monoculture or co-culture (Figure 7D and Figure S2). Moreover, the immunofluorescence of α-sarcomeric actinin and Myh3 revealed not fully organized sarcomere structures in both types of constructs (Figure 7E).

## 4. Discussion

In this study, we sought to up-scale the size of disc-shaped skeletal muscle engineered tissue from 8 mm to 50 mm in diameter while maintaining a relevant thickness of 3 mm. We adapted a previously established in vitro protocol for the one-step proliferation and differentiation of mouse SkMb co-cultured with human SVF cells. Next, we successfully demonstrated the superior vascularization and innervation potential of engineered skeletal muscle tissues co-cultured with SVF cells upon implantation into a subcutaneous pocket in nude rats.

The ratio of SkMbs to SVF cells in the present study was the same as what we previously published for the generation of small-sized engineered constructs [44]. However, the up-scaling process led to a less uniform distribution of the SVF cells throughout the construct. Following 11 days of in vitro culture, the even distribution of SVF cells was mostly found at the center and the middle of the construct compared to the peripheral areas. This lack of uniformity in the cell distribution can be explained by the fact that they were seeded in a manual and static fashion. Contrarily, cell seeding in small-sized engineered tissues was supported by bi-directional flow-based perfusion, ensuring a more homogeneous distribution [59]. The upscaled manufacturing process was also adapted in terms of in vitro culture time. While previously generated small-scaled constructs were cultured for 6 days, here we prolonged it up to 11 days. This led to significantly higher levels of maturation and a higher fusion index, thus contributing to the formation of more mature and complex muscle-like tissues. Besides for the standard differentiation and maturation parameters, in this study we also evaluated the hydraulic permeability of the entire upscaled engineered constructs as an overall index of uniformity of tissue compaction and ECM deposition. Within our measurement setting, we obtained hydraulic permeability for a cell free collagen scaffold of 1.29 × 10^−8^ m^2^ (1.29 × 10^−11^ m^4^/Ns), resulting in values close to previous studies on the permeability of collagen type I sponges [54]. Permeability was measured when scaffolds were perfused with culture medium at 37 °C and assuming the viscosity of the medium and water to be very close [53]. As expected, the hydraulic permeability of the scaffolds correlated with the size and structure of the pores within the scaffold [52] decreasing during the time in culture, confirming the formation of a more complex and mature tissue-like ECM.

As we previously demonstrated [44], co-culture of SkMbs with SVF cells does not impair their capacity of fusion into myotubes, calculated by the index of fusion, neither their overall mechanical function, evaluated by the force of contraction. However, similar to what was observed in small-scaled engineered tissues, SVF cells impaired the maturation of SkMbs, evaluated by quantification of the maturation index. Consistently, similar results were observed in another study, in which 2D monolayer co-cultures of mouse SkMbs and rat SVF cells were performed at ratios from 1:1 to 1:5. While SVF cells supported SkMb proliferation, the index of maturation was reduced, mostly through the SVF-released secretome [60]. Despite using higher ratios of SkMb to SVF cells, and culture under dynamic conditions, our findings on the impaired index of maturation may be explained by a similar effect to what El-Habta and colleagues described.

The force of contraction generated by our large-scale engineered skeletal muscle resulted in being one order of magnitude smaller than what has been obtained by other groups working on engineered muscle bundles [61]. The here-generated muscle-like tissues lack the formation of myofibers with full organized sarcomeres. Altogether, our findings reveal that the level of maturation of our engineered constructs still needs to be improved. One of the major obstacles to the complete fusion of the myoblasts into aligned myofibers and their progression in the formation of sarcomeres may be related to the use of a porous scaffold such as a collagen sponge. It requires a longer time to be remodeled in order to allow complete cell fusion and elongation. The further maturation of myoblasts into aligned myofibers could be achieved in a variety of ways, for example, by further extending the culture time or increasing the initial SkMb density in order to promote cell–cell contact and fusion, thanks also to the degradation of the porous collagen scaffold. In addition, physical cues could also be implemented during the in vitro culture, supplying, for example, electrical or mechanical stimulations [62]. Increasing the time and changing the site of the implantation (orthotopic model) could also contribute to the further maturation of the patch in vivo thanks to higher degradation of the scaffold and to exposure to proper physiological cues. Induction of vascularization upon in vivo implantation has traditionally been targeted by co-culturing parenchymal cells with endothelial cells [63], fibroblasts [64], or MSCs [62] in combination with microfabrication techniques such as electrospinning [65,66]. However, such studies aimed at vascularizing cell sheets or small-scale constructs, which was far from achieving tissues of a clinically relevant size. One key aspect that sets this study apart is the unique approach of combining murine-derived skeletal muscle myoblasts and human adipose-derived SVF cells in upscaled skeletal muscle-engineered tissue. Adipose-derived SVF, consisting of a heterogenous population rich in adult MSCs, but also abundant in vascular progenitor and mature cells, is an ideal candidate to promote vascularization without implying complex bio-fabricating processes [67]. Sampling of SVF comes along with limited donor site morbidities and high cell yields after isolation, making it ideal as an autologous cell source for pro-angiogenic tissue engineering purposes. Nevertheless, the total amount of SVF cells needed to generate one single upscaled-engineered patch is high and challenging to obtain from a single donor. In our case, the human-derived liposuction material was obtained for the main purpose of performing surgical and plastic procedures, and not exclusively for this study. Consequently, the number of biological replicates in the co-culture experimental group was only two in order to assess the cell uniformity and the maturation aspects, being a limitation of the presented study. Moreover, the high number of SVF cells needed per patch (6.9 × 10^7^ cells) made it also unfeasible to use mouse- or rat- origin SVF cells.

Of notable relevance is that our proposed approach for the generation of clinically relevant-sized skeletal muscle-like engineered tissues also induced the ingrowth of nerve-like structures. This was demonstrated by a superior amount of large NF-positive cell structures upon in vivo implantation, mainly in constructs enriched with SVF, underlining their potential in inducing neuronal ingrowth, mostly through paracrine-mediated effects. These results are in line with other published studies in which SVF showed positive effects in several models of nerve injury through the release of neurotrophic, anti-apoptotic, and anti-inflammatory factors [68,69]. Nevertheless, despite the proximity of NF- and Myh3-positive cells, NMJ structures were not observed in both monoculture and co-culture experimental groups. To further improve this aspect, the supplementation of motoneurons and the application of electrical stimulation may be considered. Many groups indeed have exploited the possibility of pre-innervating muscle-like engineered tissues, mainly by co-culture with neuronal cells or induced pluripotent stem cell-derived motoneurons, which have resulted in functional NMJ integration [37,70,71,72,73]. The scaling-up process (in terms of size and, in particular, tissue thickness) still remains an open challenge. In this regard, the in vivo implantation of 8 mm-diameter constructs instead of the entire 50 mm-diameter tissue, together with the fact of relying on an ectopic rat model instead of a more relevant skeletal VML defect, could be considered a limitation of this study. However, enhancing the vascularization and innervation of 3 mm-thick engineered tissues still represents a big challenge, and our findings can be considered as a considerable step forward, especially when compared to previous studies in which more simple cell sheets or constructs with a thickness of under 3 mm were used.

Although the level of maturation of the upscaled engineered muscle tissue did not show the hallmarks of the native skeletal muscle, a clear improvement in the maturation of the SkMbs as well as the in vivo induction of vascularization and innervation showed the underlying potential of the large patch to possibly further mature following implantation at a muscle site. When taking into consideration the size of the engineered constructs, it is quite difficult to achieve a native-like grade of maturation in vitro, and only an orthotopic implantation might provide the missing cues to promote the full development into functional muscle, using the body as an in vivo bioreactor. Future studies will aim to test the feasibility of implanting the whole up-scaled engineered tissue in a relevant skeletal muscle defect model with matching cell species.

In conclusions, this study successfully upscaled the size of skeletal muscle-engineered tissue while maintaining relevant thickness and demonstrated superior vascularization and innervation potential upon implantation. However, further improvements are needed to achieve complete maturation of the muscle-like tissues. The use of adipose-derived SVF cells and the induction of neurotrophic effects show promise for enhancing vascularization and innervation, highlighting the potential for future studies in relevant skeletal muscle defect models.

## Figures and Tables

**Table 1:** Highly organized nerve-like structures. Detection of nerve-like structures based on the roundish shape and on the high level of NF^+^ cell organization inside. Eight fields in each of 3 monoculture constructs and of 3 co-culture constructs were analyzed.

		Nerve-like (NL) Structure						
		NL-Structure 1		NL-Structure 2		NL-Structure 3		Total
Culture Condition	Replicate	Area µm^2^	Number NF^+^ Cells	Area µm^2^	Number NF^+^ Cells	Area µm^2^	Number NF^+^ Cells	
monoculture	A	9189.61	21.00	-	-	-	-	1
	B	-	-	-	-	-	-	0
	C	-	-	-	-	-	-	0
co-culture	A	8470.00	52.00	-	-	-	-	1
	B	2821.72	28.00	1766.60	12.00	5996.76	50.00	3
	C	2231.24	25.00	1447.16	8.00	6306.52	38.00	3

## Data Availability

Data supporting the reported results are available upon request from the corresponding author.

## Supplementary Materials

The following supporting information can be downloaded at: https://www.mdpi.com/article/10.3390/bioengineering10070800/s1. Figure S1: Macro images of the whole construct (diameter of 50 mm). Images show (A) how the constructs were divided, and different portions were dedicated to histological analyses and (B) how the center, middle, and peripheral portions were considered in the immunofluorescence image analysis. Figure S2: Representative images of mono- (A) and co-culture (B) constructs stained with Myh3 (green), NF (red), and α-BTX (white) show the absence of mature NMJ upon in vivo implantation. Scale bar = 20 μm.
