# Supplementary material for: Upscaled Skeletal Muscle Engineered Tissue with In Vivo Vascularization and Innervation Potential
Source: Bioengineering (Basel). 2023 Jul 4;10(7):800. doi: 10.3390/bioengineering10070800 (PMC10376693; doi:10.3390/bioengineering10070800)
Supplement: Supplementary file 1 [file bioengineering-10-00800-s001.zip › bioengineering-2414333-supplementary.pdf]

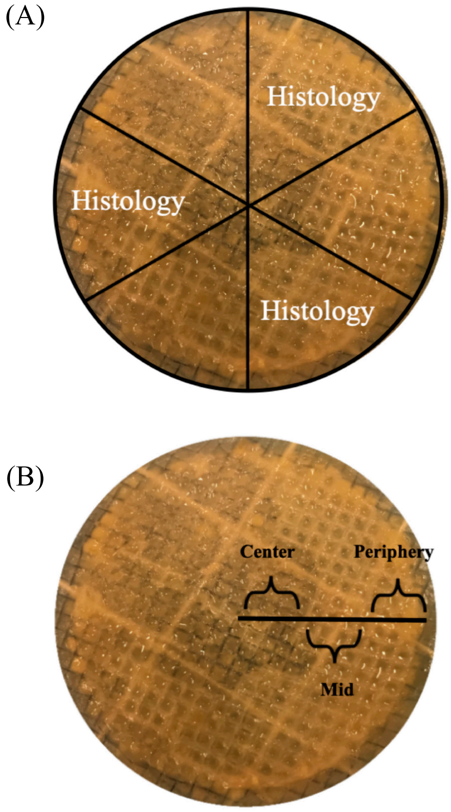

Figure S1. Macro images of the whole construct (diameter of 50 mm). Images show (**A**) how the constructs were divided, and different portions were dedicated to histological analyses and (**B**) how the center, middle, and peripheral portions were considered in the immunofluorescence image analysis.

(A)

(B)

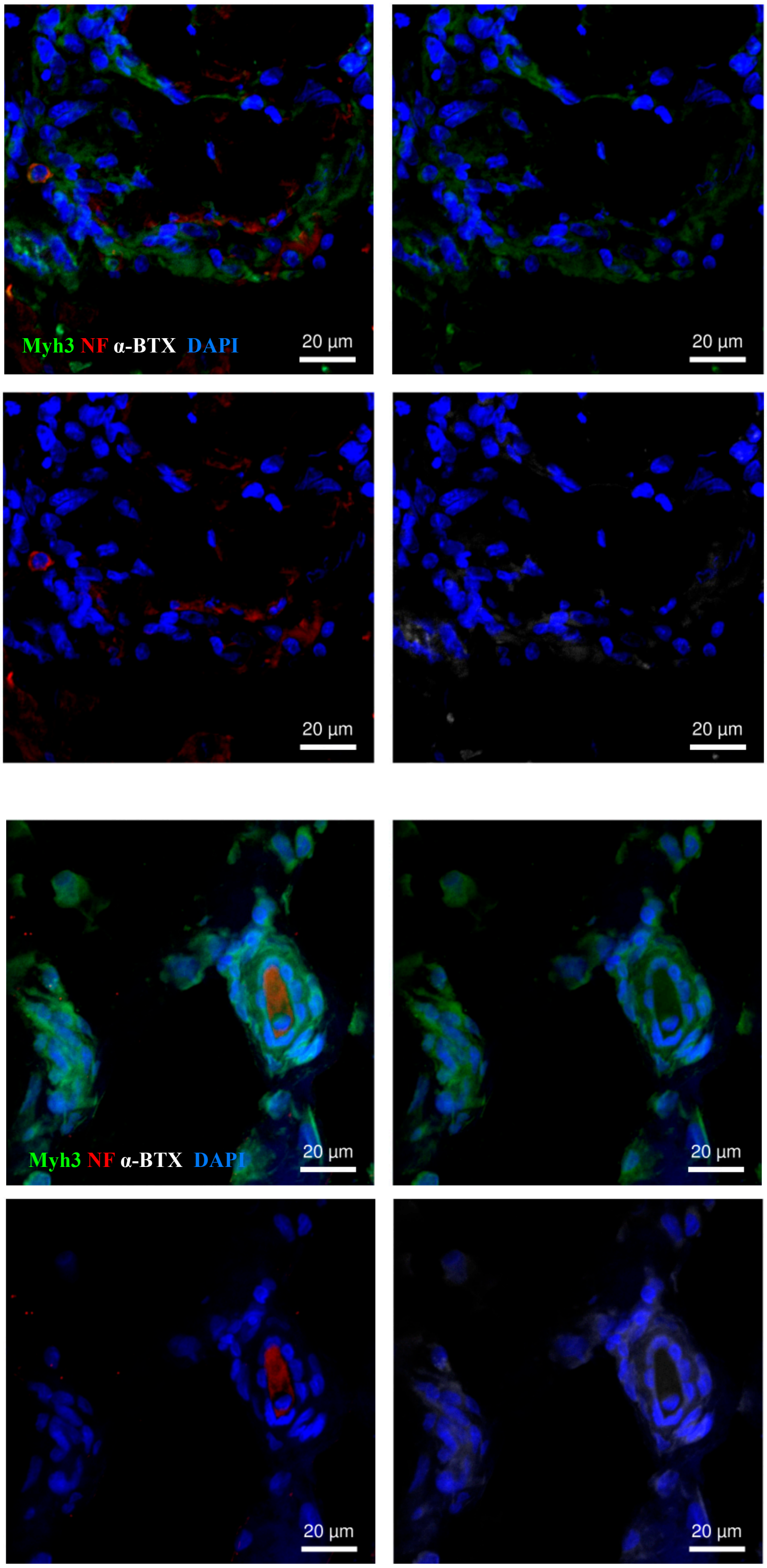

Figure S2. Representative images of mono- (**A**) and co-culture (**B**) constructs stained with Myh3 (green), NF (red), and α-BTX (white) show the absence of mature NMJ upon *in vivo* implantation. Scale bar = 20 µm.
